# Morphological Characteristics and Clinical Significance of Different Types of Tumor Vessels in Patients with Stages I-IIA of Squamous Cervical Cancer

**DOI:** 10.1155/2020/3818051

**Published:** 2020-08-12

**Authors:** Marina A. Senchukova, Elena V. Makarova, Elena I. Shurygina, Nadezhda N. Volchenko

**Affiliations:** ^1^Department of Oncology, Orenburg State Medical University, 460000 Orenburg, Russia; ^2^Department of Pathology, Orenburg State Medical University, 460000 Orenburg, Russia; ^3^Department of Pathology, P. A. Hertzen Moscow Oncology Research Center, National Medical Research Center of Radiology, 125284 Moscow, Russia

## Abstract

The determination of factors associated with progression of cervical cancer is important, both for a recurrence risk assessment and for determining optimal treatment tactics. Previously, we showed the prognostic value of different types of tumor microvessels (MVs) in gastric and breast cancer. The object of this research was to study the morphology and clinical significance of different tumor microvessels in early cervical cancer. A total of 65 archived paraffin blocks of patients with I-IIA stages of squamous cervical cancer were investigated. Samples were stained with Mayer hematoxylin and immunohistochemically using antibodies to CD34, podoplanin, HIF-1a, and Snail. The eight types of tumor MVs differed in morphology were identified. It was established that only the dilated capillaries (DСs) with weak expression of CD34, the contact type DCs, the capillaries in tumor solid component, and the lymphatic vessels in the lymphoid and polymorphic cell infiltrates of tumor stroma are associated with clinical and pathological characteristics of early cervical cancer. Preliminary results also suggest that a combination of fragmentation in tumor solid component and the contact type DCs may predict a recurrence of early cervical cancer. Given the small number of cervical cancer recurrences, the predictive significance of the described markers requires a more thorough examination.

## 1. Introduction

Cervical cancer is an urgent worldwide public health problem [1]. In 2018, about 570,000 cases of cervical cancer were identified, and 311,000 patients died from the disease. It ranks as the fourth most frequently diagnosed cancer and the fourth leading cause of cancer death in women [2, 3].

It should be noted that the diagnosis of cervical cancer in early stages is crucial for successful treatment. However, cervical cancer is characterized by an aggressive course and the detection of this pathology at an early stage does not always guarantee a favorable result. In this regard, the determination of factors associated with the progression of cervical cancer is important, both for an accurate assessment of the relapse risk of the disease and for determining the optimal tactics for treatment. Currently, the assessment of the relapse risk in early cervical cancer is based mainly on the clinical characteristics of the disease, such as age, African-American ethnicity, human papillomavirus 18 infection, histology, grade, deep cervical stromal invasion, tumor size over 2 cm, lymphovascular space invasion, nodal metastases, microscopic tumor in uterine parametrial tissues, positive surgical margins, and type of surgery [4–7]. The importance of such assessments is related to the requirement to select the patients in whom organ-preserving operations (trachelectomy) may be safe [4–6], as well as for the selection of patients requiring adjuvant therapy [8]. Given the risk of serious side effects from radiation therapy and chemotherapy, such studies are undoubtedly of interest [9–13].

Angiogenesis is one of the critical processes required for tumor growth, invasion, and metastasis [14–16]. It has been found that the activation of angiogenesis mechanisms is associated with the hypoxic microenvironment of tumor cells and, in particular, with an increase in the level of hypoxia-inducible factor-1 (HIF-1). It is known that HIF-1 is one of the key transcription factors responsible for the regulation of gene expression during hypoxia and ischemia [17, 18]. An increase in the expression level of HIF-1*α* leads to the metabolic reprogramming of tumor cells, enabling them to avoid hypoxic conditions, via invasion and metastasis, and also to improve oxygen availability, via angiogenesis and neovascularization [18–20].

We believe that when studying the role of angiogenesis in tumor progression, it is important to consider two points, as follows:

1. Structural and functional inferiority of tumor vessels leading to the deterioration of oxygen diffusion through their walls [21–26]
2. Heterogeneity of tumor vessels in origin, morphology, degree of maturity, and sensitivity to drugs [27–32]

Given these two points, it is logical to assume that the qualitative and quantitative features of tumor vessels can directly affect the severity of hypoxia in tumors, the prognosis of disease, and the sensitivity of the tumor to chemotherapy and radiation therapy.

Despite the fact that the heterogeneity of tumor vessels has been confirmed by numerous studies, to date, there has been no classification of tumor vessels that takes account of both the features of their morphology and its relationship with the clinical and morphological characteristics of the pathological process and with long-term results of treatment. A number of existing classifications are based on the origin and degree of vessel maturity, as well as their relationship with the type of histopathological tumor growth patterns [26, 33, 34].

Meanwhile, we previously studied the features of different types of tumor microvessels (MVs) in gastric and breast cancer [31, 32]. As result of the study, a new classification of tumor MVs was proposed, based on both the features of their morphology and their prognostic significance. We have described five different types of tumor MVs and structures with endothelial lining: normal MVs, dilated capillaries (DCs), atypical dilated capillaries (ADCs), structures with partial endothelial lining (cavity structures of type-1), and the distinctive cellular structures in a loose fine-fibered connective tissue of the peritumoral stroma (cavity structures of type-2), in which the DCs with weak expression of CD34 were present.

It was found that both in gastric cancer and in breast cancer, ADCs and structures with partial endothelial lining were the most significant in terms of disease prognosis. However, it is worth noting that gastric cancer and breast cancer are glandular cancers and it is not clear how the proposed classification is universal for other histological types of malignant neoplasms. In accordance with this, the aim of this study was to assess the morphological features and clinical significance of different types of tumor vessels in squamous cervical cancer.

## 2. Materials and Methods

### 2.1. Patients and Sample Collection

This pilot study was performed in accordance with the Helsinki Declaration and internationally recognized guidelines after receiving study approval from the Ethics Committee of Orenburg State Medical University (Russia, Orenburg). Sixty-five archived paraffin blocks of patients with I-IIA stages of squamous cervical cancer were retrieved from the Orenburg Regional Clinical Cancer Center tumor bank. All patients were operated on at this Center from May 2008 to March 2014.

Clinical and pathological data including age, menstrual function, comorbidity, tumor size, histology, grade and depth of tumor invasion, lymph node status, type of surgery, and the presence of adjuvant therapy were retrieved from the routine reports. Tumor size and lymph node status were categorized according to the TNM classification of malignant tumors (the 7th edition) and FIGO (2009). The privacy of patients was protected by coding of data according to the privacy regulations of the Orenburg Regional Clinical Oncologic Center (Russia, Orenburg).

None of the patients included in the study underwent preoperative chemotherapy or radiation therapy. Moreover, they did not receive steroids, nonsteroidal anti-inflammatory drugs, or antihistamines and had no significant comorbid pathologies in decompensation stage. The baseline patient's clinicopathological and treatment information is shown in Table 1.

In accordance with domestic protocol, adjuvant radiotherapy was prescribed to 29 patients with IB-IIA stages of cervical cancer who had at least two of the three risk factors (tumor size more than 4 cm, the presence of lymphovascular invasion, and more than one-third of stromal invasion). These patients underwent external beam radiotherapy (45 Gy) and brachytherapy (25 Gy). Three patients (one was with IB, and two patients were with IIA stages) underwent adjuvant chemotherapy due to the presence of contraindications to radiation therapy (they had postoperative inflammatory changes in the pelvis). In 5 (7.7%) patients, in the period from 5 to 43 months after completion of treatment, the recurrence of cervical cancer was diagnosed. Of the five patients with recurrence of cervical cancer, four patients with stage IB underwent adjuvant radiation therapy and one patient with stage IA was without adjuvant treatment. Local recurrence was detected in 2 patients, systemic recurrence in 2 patients, and local and systemic relapse in 1 patient. The median follow-up was 7.5 years.

### 2.2. Pathology

Sections (4 mm) were cut from the formalin-fixed paraffin embedded blocks. One section was stained with Mayer's hematoxylin and eosin (H&E). Histological slides were studied by light microscopy (Levenhuk D740T digital microscope, connected to a 5.1 MP camera, Russia). In the samples, the following indicators were evaluated by the visual analog way using a 200x magnification:

- The severity of lymphoid infiltration in tumor stroma (no and mild, the presence of weak diffuse infiltration or small focal infiltrates; severe, the presence of massive focal infiltrates)
- The presence of DCs in the loose, fine-fibered connective tissue of the peritumoral stroma (none and single, no more than two in the field of view; multiple, more than two in the field of view)

### 2.3. Immunohistochemistry

For immunohistochemistry (IHC), 4-*µ*m sections were dewaxed and hydrated. For antigen retrieval, the sections were boiling for 20 min in citrate buffer (pH6) using an antigen retrieval PT module (Thermo Fisher Scientific Inc., Waltham, MA, USA). Endogenous peroxidase activity was blocked with 30 mL/L hydrogen peroxide solution. Sections were then stained with the following antibodies: MAB959Hu21 monoclonal antibody to cluster of differentiation 34 (CD34), 1 : 100 dilution (Cloud-Clone Corp®, Texas, USA); PAC719Hu01 polyclonal antibody to podoplanin (PDPN), 1 : 50 dilution (Cloud-Clone Corp®, Texas, USA); PAK089Hu01 polyclonal antibody to Snail homolog 1 (SNAI1), 1 : 100 dilution (Cloud-Clone Corp®, Texas, USA); PAA798Hu02 polyclonal antibody to hypoxia-inducible factor-1 alpha, 1 : 200 dilution (Cloud-Clone Corp®, Texas, USA). The staining procedure was performed according to the manufacturers' protocols using an Autostainer 480 (Thermo Fisher Scientific Ltd., Vantaa, Finland). The visualization system included NovolinkTM Polymer Detection Kit (Leica Biosystems, Newcastle, UK). For the negative control sections, primary antibodies were replaced with phosphate-buffered saline and processed in the same manner.

The following indicators were evaluated in the samples:

- Microvessel density (MVD) was assessed in accordance with the international consensus on the methodology and criteria for quantitative evaluation of angiogenesis in human solid tumors [35]. MVD was determined by counting the number of CD34-positive and podoplanin-positive normal microvessels (MVs) in five high-power (x400) fields in the selected “hotspot” areas, and the mean values of vessel counts were obtained. A single, countable microvessel was defined as any brown-stained endothelial cell (or cluster) clearly separated from the adjacent microvessels.

The number of different DCs, ADCs, and structures with partial endothelial lining was determined using a 200x magnification:

- The absolute number of different DCs and structures with partial endothelial lining was estimated in three fields of view in the selected “hotspot” areas, and the mean values of vessel counts were obtained
- The relative number of different DCs, ADCs, and structures with partial endothelial lining was calculated using a visual analog method (none, single, and multiple, using 65 percentiles)

The following pathomorphological characteristics of tumor stromal and parenchymal component were evaluated:

- The presence of tumor emboli in CD34-positive and podoplanin-positive vessels (presence or absence).
- The presence of fragmentation in the tumor solid component (presence or absence). The presence of fragmentation was evaluated positively when fibroblast-like tumor cells with nuclear expression of HIF-1*α* and Snail were detected in the solid component of the tumor.

All sections were carefully and completely scanned by two of the authors (EM and ES) without knowledge of the clinical and pathological data.

### 2.4. Statistics

Statistical analysis was performed using the Statistica 10.0 software. The MVD and absolute number of DCs and structures with partial endothelial lining were demonstrated as mean ± SD. The normality of distribution was evaluated using the Kolmogorov–Smirnov test. Kruskal–Wallis or Mann–Whitney U nonparametric tests were used to compare the value of the quantitative and categorical data. The correlations between different data were evaluated using nonparametric Spearman's rank correlation or gamma correlation. Chi square tests were carried out to analyze the difference of distribution among the categorized data. The survival was analyzed by the Kaplan–Meier method. The logrank test was used to compare survival curves between subgroups of patients. A value of *p* < 0.05 was considered statistically significant. The results and discussion may be presented separately, or in one combined section, and may optionally be divided into headed subsections.

## 3. Results

### 3.1. Morphological Features of Different Types of Tumor Vessels in Squamous Cervical Cancer

Based on the previously proposed classification of tumor MVs [32] in cervical cancer, the following types of tumor vessels were identified: normal MVs, DCs with normal endothelial lining, DCs with a weak expression of CD34, contact type DCs, MVs in the solid component of the tumor, ADCs, structures with partial endothelial lining, and lymphatic capillaries in the lymphoid or polymorphic cell infiltrates.

#### 3.1.1. Normal Microvessels (MVs)

**Figure 1:**
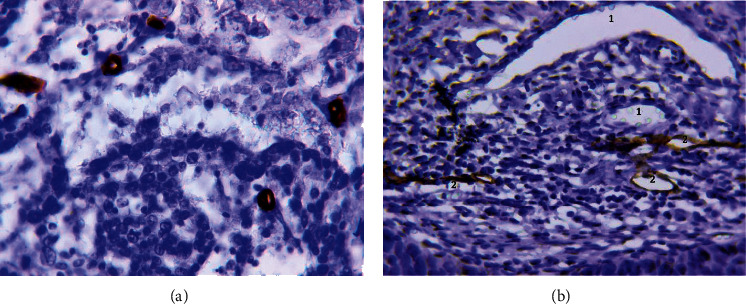
Normal microvessels. (a) Normal microvessels. Immunoperoxidase staining with antibody to CD34, x800. (b) Normal microvessels: blood capillaries (1); lymphatic capillaries (2). Immunoperoxidase staining with antibody to podoplanin, x800.

These vessels were detected as capillaries with a diameter of 5–40 *μ*m (Figure 1). In most cases, the vessels of this type had clear, even contours. Their endothelial lining was identified by cells with a flat hyperchromic nucleus. The expression of the CD34 marker in the cell cytoplasm was uniform and intense (Figure 1(a)). Capillaries with a diameter of 5–40 *μ*m were also detected when stained with antibodies to podoplanin (Figure 1(b)).

The MVD of CD34-positive normal MVs was 9.1 ± 3.4 (from 4.2 to 17.1 in the field of view, median 8.2), and the MVD of podoplanin-positive normal MVs was 3.3 ± 1.5 (from 1.0 to 9.8 in the field of view, median 3.0). The difference in the density of blood and lymphatic MVs was statistically significant (*p* < 0.0001).

*Dilated Capillaries (DCs)*. The vessels of this type had a diameter of 40 microns or more and were usually rounded or oval in shape. The expression of the CD34 marker in the cytoplasm of endothelial cells was uniform, and the vessel contours were clear and even. Depending on the characteristics of the vessels, three types of DCs were noted.

#### 3.1.2. DCs with a Normal Endothelial Lining Formed by Cells with Flattened, Hyperchromic Nuclei

**Figure 2:**
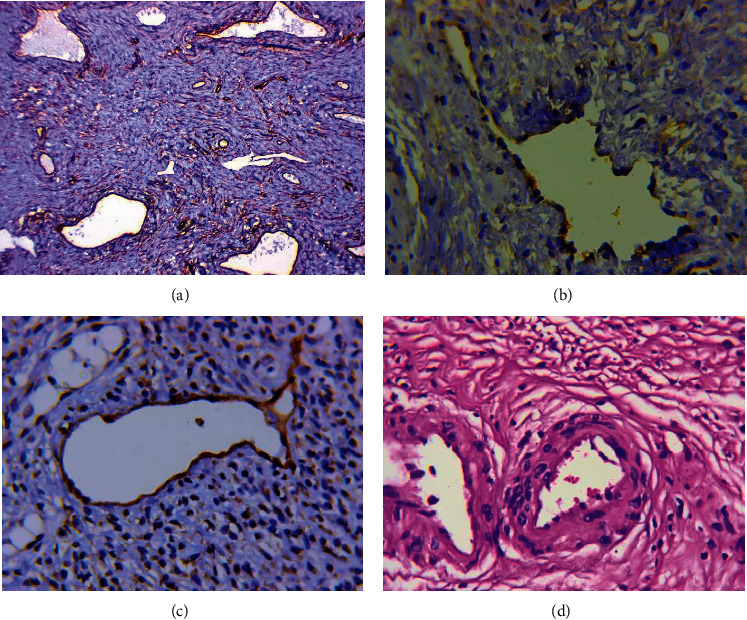
Dilated capillaries with a normal endothelial lining. (a) Dilated capillaries of regular oval shape. Immunoperoxidase staining with antibody to CD34, x800. (b) Dilated capillary of irregular angular shape. Immunoperoxidase staining with antibody to CD34, x800. (c) Dilated lymphatic capillary with a normal endothelial lining. Immunoperoxidase staining with antibody to podoplanin, x800. (d) Dilated capillaries with perivascular sheaths. H&E staining, x800.

Among the described vessels, both regular oval shape vessels (Figure 2(a)) and vessels of irregular, angular shapes (Figure 2(b)) were observed. Both erythrocytes and leukocyte cells were detected in their lumen. The density of these vessels was 5.1 ± 2.4 (from 0 to 14 in the field of view, median 5.0). In 35.4% of cases, there were multiple DCs (5.5 or more in the field of view, 65 percentile). Some vessels of this type had been stained by antibodies to podoplanin (Figure 2(c)). Dilated lymphatic capillaries were located mainly in the peritumoral stroma. In addition, some DCs had a characteristic perivascular sheath (Figure 2(d)). Such perivascular sheaths were revealed in 92.2% of the examined cervical cancer samples. Podoplanin expression was not observed in these vessels.

#### 3.1.3. DCs with a Weak Expression of CD34

**Figure 3:**
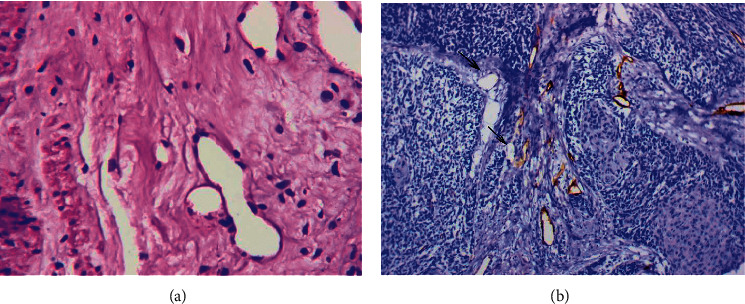
Dilated capillaries with a weak expression of CD34. (a) Dilated capillary of regular shape located in the loose, fine-fibered connective tissue of the peritumoral stroma. H&E staining, x800. (b) A weak expression of CD34 in the dilated capillaries (arrows) located in the loose, fine-fibered connective tissue of the peritumoral stroma. Immunoperoxidase staining with antibody to CD34, x200.

In squamous cervical cancer, the single vessels of this type were detected in 21 cases (32.3%), the multiple in 4 cases (6.2%), and the vessels were absent in 40 (61.5%). The DCs had a regular shape and were observed mainly in the loose, fine-fibered connective tissue of the peritumoral stroma; this had a characteristic cellular structure and was rich in cells with large, pale nuclei with a fine-netted chromatin structure (Figure 3(a)). In these vessels, the endothelial lining was also marked by cells with large, pale nuclei having a fine-netted chromatin structure. The CD34 marker was often weakly expressed in these cells (Figure 3(b)), and the expression of podoplanin was not observed, which indicates that the vessels of this type are blood vessels.

#### 3.1.4. DCs of “Contact Type”

**Figure 4:**
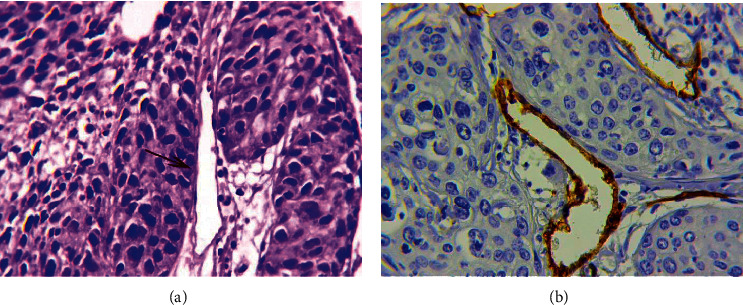
Dilated capillaries of “contact type”. (a) Dilated capillary of contact type (arrow). H&E staining, x800. (b) Dilated capillaries of contact type. Immunoperoxidase staining with antibody to CD34, x800.

A distinctive feature of these vessels was that their wall was in direct contact with tumor cells (Figure 4). The connective tissue layer between them was not determined (Figure 4(a)). Thin-walled vessels located in the intratumoral stroma were identified in 13 (20.3%) cases. The average diameter of contact type DCs was 48.7 ± 10.5 *μ*m, and the expression of the CD34 marker was uniform, with a medium intensity. The contours of the vessels were clear and even (Figure 4(b)). Despite the fact that the contents in the lumen of contact-type DCs were most often absent or presented by leukocytes, no podoplanin expression was noted in them, indicating that these vessels were blood ones.

#### 3.1.5. Capillaries in the Solid Tumor Component

**Figure 5:**
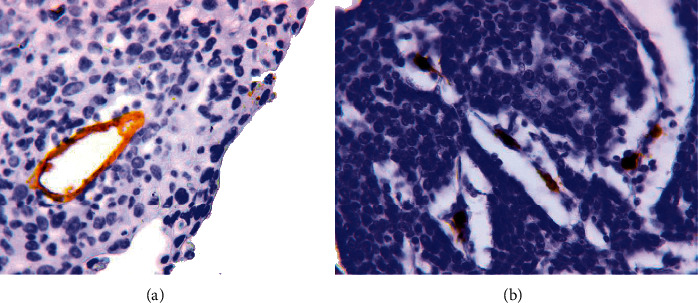
Capillaries in the solid tumor component. (a) Capillary in the solid tumor component directly in contact with tumor cells. Immunoperoxidase staining with antibody to CD34, x800. (b) Capillary in the solid tumor component with retraction of the endothelium from tumor cells. Immunoperoxidase staining with antibody to CD34, x800.

The MVs located in the tumor solid component were detected in squamous cervical cancer (Figure 5). The vessels had clear, even contours and an even expression of CD34. Podoplanin expression was not observed in these vessels. We distinguished two types of these vessels:

- *Capillaries Directly in Contact with Tumor Cells* (Figure 5(a)). These vessels were detected in 35 (54.8%) of the studied samples. The average diameter of these vessels was 64.3 ± 12.7 *μ*m, and the contents in the lumen of the vessels were most often absent or were presented by erythrocytes.
- *Capillaries with Retraction of the Endothelium from Tumor Cells.* These vessels were identified as collapsed capillaries with a linear form. The cytoplasm of the endothelial cells intensively expressed the marker CD34 (Figure 5(b)). These vessels were detected in 26 (41.7%) of the studied samples. Moreover, in 12 (18.8%) cases, both types of the described vessels were observed in the solid component of the tumor, with and without retraction of vessel walls from the tumor cells.

#### 3.1.6. Atypical Dilated Capillaries (ADCs)

**Figure 6:**
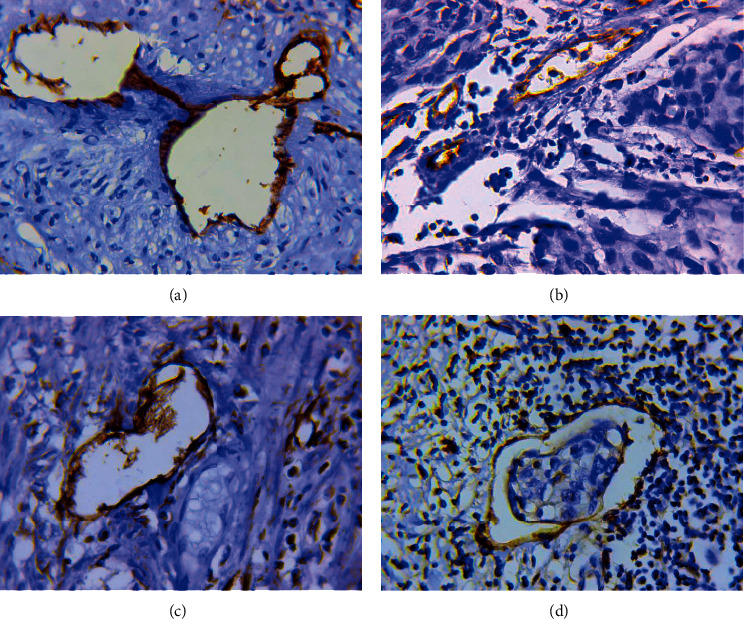
Atypical dilated capillaries. (a) Atypical dilated capillary in the peritumoral stroma. Immunoperoxidase staining with antibody to CD34, x800. (b) Atypical dilated capillary with CD34-positive cells in the lumen. Immunoperoxidase staining with antibody to CD34, x800. (c) Dilated lymphatic capillary with a chaotic arrangement of the endothelial cells. Immunoperoxidase staining with antibody to podoplanin, x800. (d) Dilated lymphatic capillary with a chaotic arrangement of the endothelial cells and a large complex of tumor cells in the lumen. Immunoperoxidase staining with antibody to podoplanin, x800.

As previously described [31, 32], a critical difference between ADCs and other types of MVs existed in the chaotic arrangement of the endothelial cells that were irregular in shape (Figure 6). The cytoplasm of the lining cells was unevenly stained by CD34 and had an uneven surface with a number of protuberances, so that the contours of the vessels seemed to be indistinct (Figure 6(a)). The density of ADCs was 1.9 ± 1.9 (from 0 to 9.7 in the field of view, median 1.5). Multiple ADCs (2 or more in the field of view, 65 percentile) were noted in 35.5% of cases. In cervical cancer, vessels of this type were significantly more frequently observed in the peritumoral than in the intratumoral stroma. As in gastric and breast cancer, tumor emboli and CD34-positive cells not associated with the vessel wall were often observed in their lumen (Figure 6(b)). Some ADCs exhibited positive staining with antibodies to podoplanin (Figure 6(c)). Almost all vessels with large complexes of tumor cells in their lumen were lymphatic vessels (Figure 6(d)).

#### 3.1.7. The Structures with a Partial Endothelial Lining

**Figure 7:**
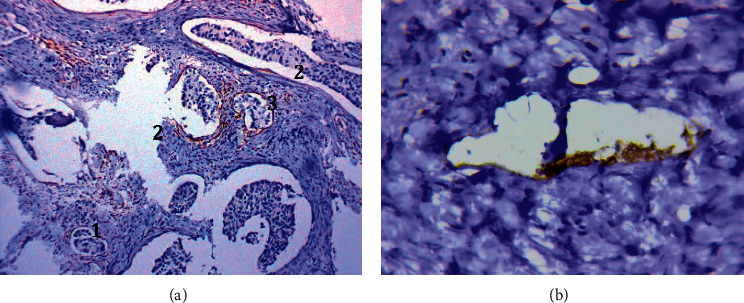
Structures with partial endothelial lining. (a) Structures with partial endothelial lining associated with the retraction of tumor cells from the underlying stroma: phenomenon of tumor stroma retraction (1), structures with partial endothelial lining (2), and lymphatic vessel with a large tumor embolus in the lumen (3). Immunoperoxidase staining with the antibodies to podoplanin, x200. (b) Structures with partial endothelial lining which were not associated with the retraction of tumor cells from the underlying stroma. Immunoperoxidase staining with the antibodies to podoplanin, x800.

As in gastric and breast cancer, structures with partial endothelial lining were revealed in cervical cancer (Figure 7). Their density was 2.8 ± 1.8 (from 0 to 8 in the field of view, median 2.6). Multiple structures with partial endothelial lining (3.3 and more in the field of view, 65 percentile) were detected in 35.4% of samples. Their characteristic feature was the chaotic arrangement of the endothelial cells with an irregular shape, uneven contours, and an uneven expression of CD34 and podoplanin markers. Analysis showed that in cervical cancer, 3/4 of structures with partial endothelial lining were positive when stained with antibodies to podoplanin. A more detailed analysis made it possible to distinguish two types of described structures:

- *Structures with Partial Endothelial Lining Associated with the Retraction of Tumor Cells from the Underlying Stroma* (Figure 7(a)). In the lumen of these structures, large or small complexes of tumor cells have always been observed. In most cases, the structures were detected in peritumoral stroma and their endothelial lining was positive when stained with antibodies to podoplanin. When their present, the structures without endothelial lining and vessels with complexes of tumor cells in their lumen were also often observed in the tumor stroma. We believe that structures without endothelial lining correspond to the previously described phenomenon of tumor stroma retraction [36].
- *Structures with Partial Endothelial Lining Which Were Not Associated with the Retraction of Tumor Cells from the Underlying Stroma* (Figure 7(b)). These structures were located mainly in the peritumoral stroma and often had a linear or irregular shape. We believe that the formation of these structures may be associated with edema and inflammatory changes in the surrounding tissues.

#### 3.1.8. Lymphatic Vessels in Lymphoid and Polymorphic Cell Infiltrates of Tumor Stroma

**Figure 8:**
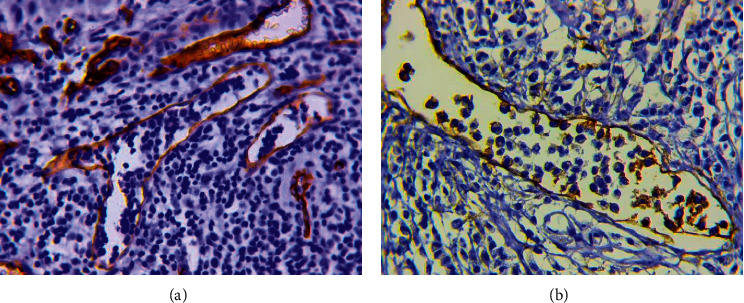
Lymphatic vessels in lymphoid and polymorphic cell infiltrates of tumor stroma. (a) Clump of lymphocytes in the lumen of thin-walled capillary located in the focal lymphoid infiltrate. Immunoperoxidase staining with the antibodies to CD34, x800. (b) Leukocyte cells in the lumen of thin-walled capillary. Some cells with fragmentary (point) expression of podoplanin. Immunoperoxidase staining with the antibodies to podoplanin, x800.

In 37 (56.9%) samples, lymphatic vessels were detected in the lymphoid and polymorphic cell infiltrates of tumor stroma; these vessels differed in the following features (Figure 8):

- The vessels had a very thin, sometimes slightly noticeable endothelial lining (Figure 8(a)).
- Lymphocytes and other leukocyte cells were often observed in their lumen (Figure 8(b)). Accumulations of cells with fragmentary (point) expression of markers and the presence of ordinary lymphatic capillaries were often observed in lymphoid infiltrates, which probably reflects different stages of the formation of lymphatic vessels of this type.

The described vessels and structures were usually not visualized when stained with Mayer hematoxylin and eosin.

**Figure 9:**
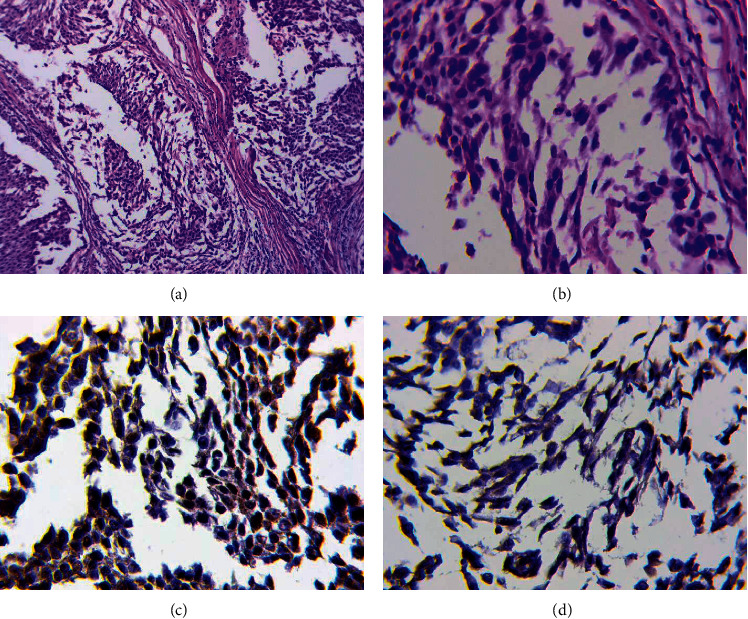
The fragmentation phenomenon in the tumor solid component. (a) The phenomenon of fragmentation in the tumor solid component. H&E staining, x200. (b) Separate fibroblast-like cells in the tumor solid component. H&E staining, x800. (c) Nuclear expression of HIF-1a in separate fibroblast-like cells in the tumor solid component. Immunoperoxidase staining with the antibodies to HIF-1a, x800. (d) Nuclear expression of Snail in separate fibroblast-like cells in the tumor solid component. Immunoperoxidase staining with the antibodies to Snail, x800.

*(1) The Phenomenon of Fragmentation in Tumor Solid Component (Figures 9(a) and 9(b))*. It should be noted that one of the interesting results of this study was the identification of the fragmentation phenomenon in the tumor solid component. The fragmentation phenomenon has been defined as the appearance of separate fibroblast-like cells with nuclear expression of HIF-1*α* (Figure 9(c)) and Snail (Figure 9(d)) in the tumor solid component. Moreover, this phenomenon was associated with some types of tumor vessels as well as with clinical characteristics and prognosis of early cervical cancer.

*(2) The Clinical Significance of Different Types of Tumor Vessels in Squamous Cervical Cancer*. It was found that in early cervical cancer, the disease recurrence was associated with age (*p* = 0.004), histology (gamma = 0.802; *p* = 0.001), the depth of tumor invasion (gamma = 0.705; *p* = 0.002), the phenomenon of fragmentation in the tumor solid component (gamma = 0.786; *p* = 0.0003), and the presence of tumor emboli in CD34-positive vessels (gamma = 0.832; *p* = 0.00003) and podoplanin-positive vessels (gamma = 0.968; *p* = 0.0003). The recurrence of cervical cancer was observed significantly more often in patients who were under 35 years of age (*p* = 0.02), had squamous cell keratinous carcinoma (*p* = 0.03), had a depth of tumor invasion of 10 mm or more (*p* = 0.02), whose histology results showed the presence of fragmentation phenomenon (*p* = 0.01), and the presence of tumor emboli in CD34-positive vessels (*p* = 0.004) and podoplanin-positive vessels (*p* = 0.009). The frequency of cervical cancer recurrence depending on the disease clinical and pathological characteristics is presented in Table 2.

The absence of correlations between stage and disease recurrence is most likely due to the fact that only patients with cervical cancer stages I-IIA were included in the study, and in whom the prognosis of the disease had been determined, firstly, by the biological characteristics of the tumor and sensitivity to adjuvant therapy. Thus, considering the above, the clinical significance of different types of tumor MVs and structures with partial endothelial lining was evaluated in line with these prognostic factors.

The analysis showed that DCs with a weak expression of CD34, “contact type” DCs, capillaries in the tumor solid component, and lymphatic vessels in the lymphoid and polymorphic cell infiltrates of the tumor stroma are the most significant factors in terms of cervical cancer prognosis.

The gamma correlation coefficient test (gamma) showed that the presence of DCs with a weak expression of CD34 correlated with stage (gamma = 0.511; *p*=0.0005), histology (gamma = 0.657; *p*=0.00001), grade (gamma = 0.322; *p*=0.009), the depth of tumor invasion (gamma = 0.735; *p* < 0.0001), the phenomenon of fragmentation in the tumor solid component (gamma = 0.750; *p*=0.000004), and the presence of tumor emboli in CD34-positive vessels (gamma = 0.543; *p*=0.0004) and podoplanin-positive vessels (gamma = 0.451; *p*=0.005), as well as with the cervical cancer recurrence (gamma = 0.769; *p*=0.0005). Data on the presence of DCs with a weak expression of CD34 depending on the clinical and the pathological cervical cancer characteristics and disease prognosis are presented in Table 3.

The depth of tumor invasion was 3.7 ± 3.8 mm, 6.8 ± 5.1 mm, and 12.5 ± 2.9 mm in the absence of DCs with weak CD34 expression, single and multiple ones, respectively (*p*=0.04).

The presence of contact type DCs correlated with stage (gamma = 0.641; *p* = 0.0007), histology (gamma = 0.642; *p* = 0.02), grade (gamma = 0.249; *p* = 0.05), the depth of tumor invasion (gamma = 0.700; *p* = 0.00002), the phenomenon of fragmentation in the tumor solid component (gamma = 0.578; *p* = 0.003), and the presence of tumor emboli in CD34-positive vessels (gamma = 0.686; *p* = 0.00003) and podoplanin-positive vessels (gamma = 0.563; *p* = 0.004), as well as with the cervical cancer recurrence (gamma = 0.893; *p* < 0.0001). Data on the presence of contact type DCs depending on the clinical and the pathological cervical cancer characteristics and disease prognosis are presented in Table 4.

The depth of tumor invasion was 4.1 ± 3.3 mm, 8.6 ± 5.6 mm, and 13.0 ± 4.7 mm in the absence of contact type” DCs, single and multiple ones, respectively (*p*=0.02).

The presence of the capillaries in the tumor solid component correlated with stage (gamma = 0.607; *p* = 0.0007), histology (gamma = 0.540; *p* = 0.0001), grade (gamma = 0.468; *p* = 0.0001), the depth of tumor invasion (gamma = 0.826; *p* < 0.00001), the phenomenon of fragmentation in the tumor solid component (gamma = 0.516; *p* = 0.01), and the presence of tumor emboli in CD34-positive vessels (gamma = 0.656; *p* = 0.00003), as well as with the cervical cancer recurrence (gamma = 0.739; *p* = 0.007). Data on the presence of the capillaries in the tumor solid component depending on the clinical and the pathological cervical cancer characteristics and disease prognosis are presented in Table 5.

The depth of tumor invasion was 3.4 ± 3.4 mm and 8.0 ± 5.4 mm in the absence and the presence of these vessels, respectively (*p*=0.001).

The presence of the lymphatic vessels in lymphoid and polymorphic cell infiltrates correlated with stage (gamma = 0.517; *p*=0.0005), histology (gamma = 0.538; *p*=0.0005), the depth of tumor invasion (gamma = 0.708; *p* < 0.00001), the phenomenon of fragmentation in the tumor solid component (gamma = 0.811; *p*=0.000001), and the presence of tumor emboli in the podoplanin-positive vessels (gamma = 0.762; *p*=0.0001). Data on the presence of these lymphatic vessels depending on the clinical and the pathological cervical cancer characteristics and disease prognosis are presented in Table 6.

The depth of tumor invasion was 4.3 ± 4.2 mm and 10.0 ± 4.7 mm in the absence and the presence of these vessels, respectively (*p*=0.001). No association of the number of vessels of this type with the presence of cervical cancer recurrence was revealed.

The number of structures with partial endothelial lining that was associated with the retraction of tumor cells from the underlying stroma, which was significantly more in cases when the depth of tumor invasion was 10 mm or more (*p*=0.003). No correlation of other types of tumor vessels with clinical characteristics and cervical cancer prognosis was noted.

It is important to note that the presence and quantity of the described MVs was interconnected. Moreover, “contact type” DCs correlated significantly with all types of vessels: with the presence of DCs with a weak expression of CD34 (gamma = 0.935; *p* < 0.000001), with the presence of capillaries in the tumor solid component (gamma = 0.736; *p* = 0.00005), and with the number of the lymphatic vessels in the lymphoid and polymorphic cell infiltrates of tumor stroma (gamma = 0.741; *p* = 0.00006). DCs with a weak expression of CD34 also correlated with the presence of capillaries in the solid tumor component (gamma = 0.528; *p* = 0.001) and the number of the lymphatic vessels in the lymphoid and polymorphic cell infiltrates of tumor stroma (gamma = 0.706; *p* = 0.0002). In turn, the presence of capillaries in the solid tumor component correlated with the number lymphatic vessels in the lymphoid and polymorphic cell infiltrates of tumor stroma (gamma = 0.546; *p* = 0.007).

*(3) Different Types of Tumor Microvessels and Prognosis of Stage I-IIA Cervical Cancer*. As noted above, in 5 (7.7%) patients, from 5 to 43 months after the completion of treatment, the recurrence of cervical cancer was diagnosed. Local recurrence was detected in 2 patients, systemic recurrence in 2 patients, and local and systemic relapse in 1 patient. All patients with cervical cancer recurrence died in the period from 12 to 36 months after end of treatment. Data on the 5-year relapse-free survival (RFS) depending on cervical cancer clinical and pathological characteristics and the presence of different types of tumor MVs are presented in Table 7.

For patients with early cervical cancer, it is of fundamental importance to assess the feasibility of adjuvant treatment and to predict its effectiveness. For this purpose, we compared the clinical and pathological characteristics of cervical cancer and the number of different types of tumor MVs in three groups of patients with stage IB-IIA. In group 1, there were 11 patients without radiation therapy and without relapse, in group 2, there were 21 patients who received radiation therapy and without cervical cancer recurrence, and in group 3-4, there were patients who received radiation therapy and with cervical cancer recurrence. Respectively, in groups 1, 2, and 3, there were 18.2%, 28.6%, and 75% of patients under 35 years of age (*p*=0.10); 54.6%, 47.6%, and 75% with squamous cell keratinous carcinoma (*p*=0.58); 36.4%, 47.6%, and 100% of cases with a depth of tumor invasion of 10 mm or more (*p*=0.09); 27.3%, 14.3%, and 75% with the presence of fragmentation phenomenon in the tumor solid component (*p*=0.04)); 54.6%, 47.6%, and 100% with the presence of DCs with a weak expression of CD34 (*p*=0.15); 9.1%, 28.6%, and 100% with the presence of “contact type” DCs (*p*=0.003); 45.5%, 42.9%, and 100% with the presence of capillaries in the tumor solid component (*p*=0.1); and 23.7%, 19.1%, and 50% of cases with the presence of lymphatic vessels in the lymphoid and polymorphic cell infiltrates of tumor stroma (*p*=0.41). Interestingly, a combination of the phenomenon of fragmentation in the tumor solid component and the presence of “contact type” DCs was observed in 3 of 4 (75.0%) patients with cervical cancer recurrence and only 1 of 32 (3.1%) patients without recurrence (*p*=0.0001).

## 4. Discussion

Сervical cancer remains one of the most important issues in modern oncology that is related with both high morbidity and mortality [2, 3]. A key condition for reducing mortality from cervical cancer is the diagnosis of the disease in the early stages. However, cervical cancer, even in the early stages, is characterized by an aggressive course, and early diagnosis does not always guarantee the success of the treatment. In this regard, the problems of accurately assessing the risk of cervical cancer recurrence and choosing the optimal treatment tactics have not lost their relevance.

It is important to emphasize that in early cervical cancer, the assessment of the risk of recurrence is of fundamental importance for deciding on the need for adjuvant therapy. Currently, this assessment is based mainly on the clinical and pathological characteristics of cervical cancer, such as age, stage, tumor size, histological type and depth of tumor invasion, positive surgical margins, the presence of metastases in the lymph nodes, the presence of parametric and perineural invasion, and some other factors [37–40]. Despite careful selection, in some patients, adjuvant therapy is not only ineffective but also leads to the development of severe complications [9, 10, 12, 13, 41–44]. In this regard, the problem of selecting patients with a low risk of disease recurrence, as well as with chemo- and radioresistant tumors, remains relevant and the search for new prognostic and predictive markers of cervical cancer has not lost its significance.

Angiogenesis is one of the key factors in tumor progression [23–26, 45, 46]. At present, assessment of its activity is considered as an important marker of disease prognosis and sensitivity to anticancer therapy [47, 48], including cervical cancer [14–16]. The participation of angiogenesis in tumor progression is due to the fact that, on the one hand, tumor vessels transport tumor cells to the lymphatic collectors and target organs. On the other hand, tumor vessels (that are defective both anatomically and functionally) are not able to adequately supply tumor cells with oxygen and nutrients. This enhances tumor hypoxia, which can lead to both necrotic changes in the tumor and to activation of epithelial-mesenchymal transformation (EMT) mechanisms that allow tumor cells to survive under conditions of hypoxia, as well as chemo- and radioresistance [17]. In addition, chemo- and radioresistance of malignant neoplasms can also be associated with vessel co-option or hijacking normal blood vessels by tumor cells [49].

It was established that the formation of new vessels is associated with the activation of various factors, amongst which a special role belongs to the vascular endothelial growth factor (VEGF), which is expressed by tumor and stromal cells. Of the 5 subtypes of the VEGF family proteins (VEGF-A, -B, -C, and -D, and placenta growth factor (PlGF)), VEGF-A is the key protein responsible for the proliferation, survival, and mobilization of endothelial progenitor cells from the bone marrow into the peripheral circulation. By binding to membrane tyrosine kinase receptors (VEGFR-1, -2, and -3), VEGF affects the development of new, and survival of immature, blood vessels [46]. Increased VEGF expression attracts monocytes and macrophages into the tumor stroma, which contributes to the activation of matrix metalloproteinases (MMPs) and cell adhesion molecules [46, 50, 51], degradation of the intercellular matrix, and initiation of the processes of invasion, metastasis, and angiogenesis [52, 53].

To evaluate the activity of angiogenesis, researchers use various markers, including VEGF, main fibroblast growth factor (bFGF), MVD, and several others. In cervical cancer, an assessment of the prognostic significance of angiogenesis activity using these markers did not allow unambiguous results [14–16, 54]. In addition, a very limited number of publications related to the study of angiogenesis features in cervical cancer have been presented in PubMed and most of which were performed more than 10 years ago. Thus, 14 studies, dated 2000–2011, were included in a meta-analysis of Zhang et al. [16]. This showed that overexpression of VEGF or VEGF-C is significantly associated with poor survival outcome in cervical cancer patients and can be used as a prognostic and predictive marker. Similar results were obtained in a meta-analysis of Sun et al. [55].

Researchers have shown that VEGF-positive expression is associated with a higher risk of lymph node metastases in cervical cancer. However, these results were observed only among Asian, Caucasian, and Chinese populations, but not among Korean or Japanese populations. A meta-analysis of Hu et al. included 13 studies dated 1997–2009 [15]. The authors noted that only MVD in the tumor, evaluated using factor VIII as an endothelial biomarker, was associated with long-term cervical cancer treatment results. When evaluating angiogenesis activity using antibodies to CD31, CD34, or CD105, correlations of MVD with cervical cancer prognosis were not revealed. In a study by Yani et al., the authors noted an increase in the level of VEGF and MVD in samples of squamous cell carcinoma compared with cervical inflammation and cervical intraepithelial neoplasia; however, there was no association of these markers with the stage of cervical cancer [56].

It should be noted that in the research mentioned above, the study of the prognostic significance of angiogenesis in cervical cancer was associated exclusively with a quantitative assessment of the expression of the various markers. Meanwhile, many modern studies indicate that vessels in tumors are heterogeneous and differ in origin, morphology, the degree of maturity, and sensitivity to anticancer therapy [57–60]. Thus, we previously studied the morphological features and clinical significance of different types of tumor vessels in gastric and breast cancer [31, 32]. We found that blood vessels differ not only in morphology, but also in clinical significance. As a result of this study, we identified 5 types of MVs and structures with endothelial lining: normal capillaries, DCs, ADCs, cavitary structures of type-1, and cavitary structures of type-2.

In breast and gastric cancer, the most significant prognostic factors were ADCs and structures with a partial endothelial lining (cavitary structures of type-1), which had similar morphological features, significantly correlated with each other (*p* < 0.00001) and were associated with the same disease characteristics. In gastric cancer, multiple ADCs and structures with partial endothelial lining were significantly more often observed at T3-4 (*p* = 0.001) and *N*2 stages (*p* = 0.001) and their presence was associated with a decrease in overall (*p* = 0.001) and relapse-free survival (RFS) (*p* = 0.0001). In breast cancer, the described vessels and structures were significantly more often observed in estrogen negative tumors (*p* = 0.03) and in the presence of tumor emboli in the vessels (*p* = 0.08). In breast cancer, there was also a correlation between the cavitary structures of type-2 and a positive HER2/neu status (*p* = 0.008). These data indirectly indicated the general mechanisms of the formation of ADCs and structures with partial endothelial lining.

In this research, the object of the study was the features of morphology and clinical significance of different types of tumor vessels in early squamous cervical cancer. All patients at the first treatment stage underwent various surgery (various hysterectomy options were performed in 93.8% of cases), 29 (44.6%) patients received adjuvant radiation therapy, and 3 (4.6%) received adjuvant chemotherapy. In 5 (7.7%) patients, in the period from 5 to 43 months after completion of treatment, the recurrence of cervical cancer was diagnosed. Given that only patients with I-IIA stages were included in the study, in whom the disease prognosis was determined primarily by the tumor biological characteristics and sensitivity to the adjuvant therapy, we first evaluated the clinical and pathological characteristics of cervical cancer associated with the development of disease recurrence. As a result of the analysis, it was found that cervical cancer recurrence was significantly more frequently observed in patients younger than 35 years (*p*=0.02), with a tumor invasion depth of 10 mm or more (*p*=0.02), in cases of squamous nonkeratinized cancer (*p*=0.03), in the presence of fragmentation phenomenon in the solid component of the tumor (*p*=0.01), as well as in the presence of tumor emboli in the blood (*p*=0.004) and lymphatic vessels (*p*=0.009). These data allowed us to evaluate the clinical significance of tumor vessels in early stage of squamous cervical cancer, taking into account not only the presence of disease recurrence, but also these prognostic factors.

A comparative analysis of the morphology of the different tumor vessels has allowed us to identify the vessels that we described earlier in gastric and breast cancer as well as to describe new vessels. In addition, we decided to change some vessel names to new ones, allowing us to more accurately characterize the features of the observed vessels and structures with endothelial lining. In particular, the cavitary structures of type-1 were renamed as the structures with partial endothelial lining, and cavitary structures of type-2 were renamed as DCs with weak expression of CD34. The use of antibodies to CD34 and podoplanin as markers made it possible to clarify the features of the morphology and localization of lymphatic vessels, to compare their number with the number of CD34-positive vessels as well as to evaluate the clinical significance of the lymphatic vessels in early cervical cancer. Summary data on the morphology of the described MVs and their correlations with clinical characteristics and prognosis of early cervical cancer are presented in Tables 8 and 9.

Analysis of the data presented in the summary tables allowed us to highlight some common features in morphology and clinical significance of different types of tumor vessels. In particular, the normal MVs and DCs with normal endothelial lining were localized both in intra- and in peritumoral stroma, were intensively stained with antibodies to CD34, and had clear, even contours. Some vessels of these types were positive when stained by antibodies to podoplanin, but the density of CD34-positive vessels was almost three times higher than the density of podoplanin-positive ones (*p* < 0.0001). No significant differences in the density of these vessels depending on the clinical and pathological characteristics and the prognosis of early cervical cancer were found.

A common morphological feature of ADCs and the structures with partial endothelial lining is that their endothelial lining was formed by chaotic located endothelial cells having an irregular shape and unevenly expressing the CD34 marker. Staining of the samples with antibodies to podoplanin demonstrated that the majority of ADCs and structures with partial endothelial lining located in the peritumoral stroma are related to the lymphatic vessels. In contrast to breast and gastric cancer, the correlations of ADCs and structures with partial endothelial lining with clinical characteristics and cervical cancer prognosis have not been established. Perhaps this is due to the fact that only patients with I-IIA stages of cervical cancer, having a favorable prognosis, were included in the study.

We previously suggested that the formation of tumor vessels can occur not only due to sprouting angiogenesis, vessel co-option, vasculogenic mimicry, intussusceptive angiogenesis, or vasculogenesis [26, 34, 61, 62], but also due to the formation of “cavitary” structures in the tumor stroma, being then lined by the endothelium and merged into the blood vessels of the organ [31, 32]. We hypothesized that there are two main mechanisms for the formation of such structures. The first mechanism can be associated with the abruption of tumor cells from their underlying foundation, and the second with the processes of formation and lysis of tumor stroma, which occur most actively at the border of tumor with the surrounding tissues.

We believe that this hypothesis is in good agreement with another assumption that tumor angiogenesis and vasculogenesis, in fact, mimic the processes of embryonic vasculogenesis [63, 64]. It is known that during embryonic vasculogenesis, the formation of interstitial canals and gaps located in the mesenchyme precedes the formation of lymphatic and blood vessels. Tumor angiogenesis is also associated with remodeling of the extracellular matrix. Under the influence of matrix metalloproteinases, cleavage of the connective tissue matrix occurs with the formation of channels for growing vessels and vasculogenous mimicry [65]. Both in embryogenesis and in tumor growth, the stroma is directly involved in formation of channels, which determine the basis and direction of newly formed vessel growth [66]. It can be assumed that the cavitary type of angiogenesis, in principle, is a variant of postnatal vasculogenesis, where the role of channels and cracks can be played the cavitary structures formed as a result of retraction of tumor cells from the underlying stroma, the processes of formation, and lysis of the tumor stroma as well as tissue stratification as a result of edema or inflammation. Whether vessels formed in this way are functional and how they can be associated with the chemo- and radioresistance of malignant neoplasms remains to be investigated.

The data in Table 9 show that only DCs with weak expression of CD34, DCs of contact type, intratumoral microvessels, and lymphatic vessels in the lymphoid and polymorphic cell infiltrates of tumor stroma were the most significant in terms of prognosis of early cervical cancer. It should be noted that DCs with weak expression of CD34 and DCs of contact type correlated with each other (*p* < 0.000001) were negative when stained by antibodies to podoplanin, and the contents in their lumen were absent or were presented by lymphoid cells. The weak staining of the endothelial cells with antibodies to CD34 and the presence of cells with large, pale nuclei having a fine-netted chromatin structure in endothelial lining may indirectly indicate that progenitor stem cells can take part in their formation. It is known that the earliest precursor cells are CD34 negative and, in the presence of relevant factors in the microenvironment, are able to differentiate into different cell lines, including into endothelial cells [67].

It can be assumed that the described vessels, creating a moderate hypoxic environment, can contribute to the survival of tumor cells due to the activation of EMT mechanisms. This assumption is indirectly confirmed by the correlation of DCs with a weak expression of CD34 with the fragmentation phenomenon in the tumor solid component (*p* = 0.001). In the presence of the fragmentation phenomenon, the tumor cells assumed an elongated fibroblast-like form and showed nuclear expression of HIF-1a and Snail. Cancer stem cells are known to represent the main pool of chemo- and radioresistant cells, and their high level in the tumor is associated with an unfavorable prognosis for a disease [68–70]. In our study, in patients receiving adjuvant radiation therapy, a combination of contact-type vessels and the fragmentation phenomenon was observed in 3 of 4 (75%) patients with cervical cancer recurrence and only in 1 out of 32 (3.1%) without cervical cancer recurrence (*p* = 0.0001). Given the small number of cervical cancer recurrences, the predictive significance of the described marker requires a more thorough examination.

Here, we also describe two new types of MVs that are correlated with prognostically significant clinical and pathological characteristics of early cervical cancer: capillaries in the tumor solid component and lymphatic vessels in the lymphoid and polymorphic cell infiltrates of tumor stroma. The mechanism of their formation and the role in tumor progression are not quite understood and require further studies.

## 5. Conclusions

Thus, the results of the study indicate that in cervical cancer, tumor vessels are heterogeneous and differ in morphology and clinical significance. The most significant factors from the point of view of early cervical cancer prognosis were DCs with a weak expression of CD34, “contact type DCs,” capillaries in the tumor solid component, and lymphatic vessels in the lymphoid and polymorphic cell infiltrates of tumor stroma. However, this study has a number of limitations related to the fact that only patients with early cervical cancer, who had a low risk of disease recurrence, were included in the study. We believe that further studies are needed to verify the assumptions made about the role of different types of tumor vessels in the progression of squamous cervical cancer as well as other types of squamous cell carcinoma.

## Figures and Tables

**Table 1:** The baseline patient's clinicopathological and treatment information.

Clinical and pathological data	*n*	%
*Age (years)*		
<35	18	27.7
35–44	15	23.1
≥45	32	49.2

*Menstrual function*		
Absence	46	70.8
Presence	19	29.2

*Comorbidity*		
Absence	8	12.3
Presence	57	87.7

*Histology*		
Squamous cell keratinous carcinoma	22	33.8
Squamous cell nonkeratinous carcinoma	43	66.2

*Grade*		
G1	49	75.4
G2	9	13.8
G3	7	10.8

*Depth of tumor invasion*		
<10 mm	44	67.7
≥10 mm	21	32.3

*T*		
Т1	61	93.8
Т2	4	6.2

*Stages*		
IA	28	43.1
IB	32	49.2
IIA	5	7.7

*Type of surgery*		
Radical hysterectomy with appendages	27	41.5
Radical hysterectomy without appendages	13	20.0
Simple hysterectomy	21	32.3
Radical trachelectomy	4	6.2

*Adjuvant radiation therapy*		
Presence	29	44.6
Absence	36	55.4

*Adjuvant chemotherapy*		
Presence	3	4.6
Absence	62	95.4

**Table 2:** The frequency of cervical cancer recurrence depending on the disease clinical and pathological characteristics.

Clinical and pathological data	Cervical cancer recurrence				*p*
	Absence		Presence		
	*n*	%	*n*	%	
*Age (years)*					
<35	4	23.5	13	76.5	0.02^*∗*^
35–44	1	3.1	31	96.9	
≥45	0	0	15	100.0	

*Stages*					
IA	1	3.6	27	96.4	0.33
IB	4	12.9	27	87.1	
IIА	0	0	5	100.0	

*Histology*					
Squamous cell keratinous carcinoma	4	18.2	18	81.8	0.03^*∗*^
Squamous cell nonkeratinous carcinoma	1	2.4	41	97.6	

*Grade*					
G1	46	95.8	2	4.2	0.14
G2	7	77.8	2	22.2	
G3	6	85.7	1	14.3	

*Depth of tumor invasion*					
<10 mm	1	2.3	42	97.7	0.02^*∗*^
≥10 mm	4	19.1	17	80.9	

*Fragmentation phenomenon*					
Absence	2	3.9	50	96.1	0.01^*∗*^
Presence	3	25.0	9	75.0	

*Tumor emboli in CD34-positive vessels*					
Absence	0	0	33	100.0	0.004^*∗*^
Presence	5	22.7	17	77.3	

*Tumor emboli in podoplanin-positive vessels*					
Absence	0	0	32	100.0	0.009^*∗*^
Presence	5	21.7	18	78.2	

*Depth of tumor invasion*	4.6 ± 4.2		12.4 ± 6.4		0.01^*∗*^

**Table 3:** The presence of DCs with a weak expression of CD34 depending on the clinical and the pathological cervical cancer characteristics and disease prognosis.

Indicators	Dilated capillaries with weakly expression of CD34				*p*
	Absence		Presence		
	*n*	%	*n*	%	
*Age (years)*					
<35	10	55.6	8	44.4	0.45
35–44	8	53.3	7	46.7	
≥45	22	68.7	10	31.3	

*Stages*					
IA	23	82.1	5	17.9	0.008^*∗*^
IB	14	43.8	18	56.2	
IIA	3	60.0	2	40.0	

*Histology*					
Squamous cell keratinous carcinoma	32	74.4	11	26.6	0.008^*∗*^
Squamous cell nonkeratinous carcinoma	8	36.4	14	63.6	

*Grade*					
G1	34	69.4	15	30.6	0.05^*∗*^
G2	4	44.4	5	56.6	
G3	2	28.6	5	71.4	

*Depth of tumor invasion*					
<10 mm	33	75.0	11	25.0	0.0007^*∗*^
≥10 mm	7	33.3	14	66.7	

*Fragmentation phenomenon*					
Absence	37	69.8	16	30.2	0.001^*∗*^
Presence	3	25.0	9	75.0	

*Tumor emboli in CD34-positive vessels*					
Absence	23	69.7	10	30.3	0.13
Presence	10	43.5	13	56.5	

*Tumor emboli in podoplanin-positive vessels*					
Absence	25	71.4	10	28.6	0.13
Presence	14	48.3	15	51.7	

*Recurrence*					
Absence	37	64.9	20	35.1	0.004^*∗*^
Presence	1	20.0	4	80.0	

**Table 4:** The presence of contact type DCs depending on the clinical and the pathological cervical cancer characteristics and disease prognosis.

Indicators	“Contact type” dilated capillaries				*p*
	Absence		Presence		
	*n*	%	*n*	%	
*Age (years)*					
<35	14	77.8	4	22.2	0.55
35–44	13	86.7	2	13.3	
≥45	24	77.4	7	22.6	

*Stages*					
IA	26	96.3	1	3.7	0.14
IB	21	65.6	11	34.4	
IIA	4	80.0	1	20.0	

*Histology*					
Squamous cell keratinous carcinoma	36	85.7	6	14.3	0.29
Squamous cell nonkeratinous carcinoma	15	68.2	7	31.8	

*Grade*					
G1	41	85.4	7	14.6	0.04^*∗*^
G2	6	66.7	3	33.3	
G3	4	57.1	3	42.9	

*Depth of tumor invasion*					
<10 mm	39	90.7	4	9.3	0.007^*∗*^
≥10 mm	12	57.1	9	42.9	

*Fragmentation phenomenon*					
Absence	44	84.6	8	15.4	0.09
Presence	7	58.3	5	41.7	

*Tumor emboli in CD34-positive vessels*					
Absence	30	90.9	3	9.1	0.009^*∗*^
Presence	13	59.1	9	40.9	

*Tumor emboli in podoplanin-positive vessels*					
Absence	30	88.2	4	11.8	0.06
Presence	20	68.9	9	31.1	

*Recurrence*					
Absence	48	85.7	8	14.3	0.0003^*∗*^
Presence	1	20.0	4	80.0	

**Table 5:** The presence of the capillaries in the tumor solid component depending on the clinical and the pathological cervical cancer characteristics and disease prognosis.

Indicators	Capillaries in the tumor solid component				*p*
	Absence		Presence		
	*n*	%	*n*	%	
*Age (years)*					
<35	8	44.4	10	55.5	0.31
35–44	21	65.6	11	34.4	
≥45	9	64.3	5	35.7	

*Stages*					
IA	21	77.8	6	22.2	0.02^*∗*^
IB	16	50.0	16	50.0	
IIA	1	20.0	4	80.0	

*Histology*					
Squamous cell keratinous carcinoma	30	78.9	12	21.1	0.02^*∗*^
Squamous cell nonkeratinous carcinoma	8	36.4	14	63.6	

*Grade*					
G1	45	91.8	4	8.2	0.0002^*∗*^
G2	6	75.0	2	25.0	
G3	2	28.6	5	71.4	

*Depth of tumor invasion*					
<10 mm	33	76.7	10	23.4	0.00005^*∗*^
≥10 mm	5	23.8	16	76.2	

*Fragmentation phenomenon*					
Absence	34	64.2	19	35.8	0.09
Presence	4	36.4	7	63.6	

*Tumor emboli in CD34-positive vessels*					
Absence	24	72.7	9	37.3	0.01^*∗*^
Presence	9	39.1	14	60.9	

*Tumor emboli in podoplanin-positive vessels*					
Absence	23	65.7	12	34.3	0.26
Presence	15	51.7	14	48.3	

*Recurrence*					
Absence	35	62.5	21	37.5	0.06
Presence	1	20.0	4	80.0	

**Table 6:** The presence of lymphatic vessels in lymphoid and polymorphic cell infiltrate depending on the clinical and the pathological cervical cancer characteristics and disease prognosis.

Indicators	Lymphatic vessels in the tumor stroma lymphoid and polymorphic cell infiltrates				*p*
	No, single		Multiple		
	*n*	%	*n*	%	
*Age (years)*					
<35	10	55.6	8	44.4	0.26
35–44	8	53.3	7	46.7	
≥45	24	75.0	8	25.0	

*Stages*					
IA	23	82.1	5	17.9	0.1
IB	17	53.1	15	46.9	
IIA	2	40.0	3	60.0	

*Histology*					
Squamous cell keratinous carcinoma	32	74.4	11	25.6	0.04^*∗*^
Squamous cell nonkeratinous carcinoma	10	45.5	12	55.5	

*Grade*					
G1	42	85.7	7	14.3	0.57
G2	7	77.8	2	22.2	
G3	5	71.4	2	28.6	

*Depth of tumor invasion*					
<10 mm	34	77.3	10	22.7	0.0004^*∗*^
≥10 mm	8	38.1	13	61.9	

*Fragmentation phenomenon*					
Absence	48	90.6	5	9.4	0.002^*∗*^
Presence	6	50.0	6	50.0	

*Tumor emboli in CD34-positive vessels*					
Absence	29	87.9	4	12.1	0.09
Presence	16	69.6	7	30.4	

*Tumor emboli in podoplanin-positive vessels*					
Absence	33	94.3	2	5.7	0.007^*∗*^
Presence	20	68.9	9	31.1	

*Recurrence*					
Absence	36	63.2	21	36.8	0.28
Presence	3	60.0	2	40.0	

**Table 7:** 5-year relapse-free survival depending on cervical cancer clinical and pathological characteristics and the presence of different types of tumor vessels.

Indicators	5-year relapse-free survival (%)	*p*
*Age (years)*		
<35	76.5	0.004^*∗*^
≥35	97.9	

*Stages*		
IA	96.4	0.26
IB-IIA	88.9	

*Depth of tumor invasion*		
<10 mm	97.7	0.02^*∗*^
≥10 mm	80.9	

*Histology*		
Squamous cell keratinous carcinoma	97.6	0.03^*∗*^
Squamous cell nonkeratinous carcinoma	81.8	

*Grade*		
G1	95.8	0.13
G2	77.8	
G3	85.7	

*Fragmentation phenomenon*		
Absence	96.1	0.01^*∗*^
Presence	75.0	

*Dilated capillaries with weakly expression of CD34*		
Absence	97.4	0.04^*∗*^
Presence	84.0	

*“Contact type” dilated capillaries*		
Absence	98.0	0.003^*∗*^
Presence	667	

*Capillaries in the tumor solid component*		
Absence	97.4	0.04^*∗*^
Presence	83.3	

*Lymphatic vessels in the tumor stroma lymphoid and polymorphic cell infiltrates*		
No, single	94.3	0.13
Multiple	81.8	

**Table 8:** Summary data on the tumor microvessel morphology in squamous cervical cancer.

	Normal microvessels	DCs with a normal endothelial lining	DCs with a weak expression of CD34	DCs of contact type	Capillaries in the solid tumor component	Atypical dilated capillaries	The structures with a partial endothelial lining	Lymphatic vessels in lymphoid and polymorphic cell infiltrates
Localization	In intra- and peritumoral stroma	In intra- and peritumoral stroma	Mainly in peritumoral stroma	Mainly in intratumoral stroma	In the solid tumor component	In intra- and peritumoral stroma	Mainly in peritumoral stroma	Mainly in peritumoral stroma
Diameter (*μ*m)	19.7 ± 10.0	101.9 ± 52.2	59.4 ± 29.6	48.7 ± 10.5	Collapsed	109.7 ± 43.9	—	85.2 ± 33.3
Shape	Regular	Regular and irregular	Regular	Regular	Linear	Irregular	Irregular	Regular
Contours	Smooth	Smooth	Smooth	Smooth	Smooth	Uneven	Uneven	Smooth
Arrangement of the endothelial cells	Normal	Normal	Normal	Normal	Normal	Chaotic	Chaotic	Normal
The severity of CD34 expression	Intensively	Intensively	Weakly	Moderately	Intensively	Intensively	Intensively	Moderately
Podoplanin staining	Some positive	Some positive	Negative	Negative	Negative	Some positive	Some positive	All positive
Content	Erythrocytes, white blood cells	Erythrocytes, white blood cells	Often absent	Absent or white blood cells	—	Absent, erythrocytes, white blood cells	Absent or tumor mass	Lymphocytes and other white blood cells
Tumor emboli in the lumen	Rarely	Rarely	Rarely	Rarely	Rarely	Often	Often	Rarely
CD34-positive cells in the lumen	Rarely	Rarely	Absent	Absent	Absent	Often	Often	Absent

**Table 9:** Summary data on the correlation of different types of tumor microvessels with clinical and morphological characteristics and prognosis of squamous cervical cancer.

	Normal microvessels	DCs with a normal endothelial lining	DCs with a weak expression of CD34	DCs of contact type	Capillaries in the tumor solid component	Atypical dilated capillaries	The structures with a partial endothelial lining	Lymphatic vessels in lymphoid and polymorphic cell infiltrates
Age	*p*=0.92	*p*=0.94	*p*=0.98	*p*=0.22	*p*=0.08	*p*=0.48	*p*=0.18	*p*=0.13
Stages	*p*=0.82	*p*=0.11	*p*=0.0005^*∗*^	*p*=0.0007^*∗*^	*p*=0.0007^*∗*^	*p*=0.36	*p*=0.48	*p*=0.0005^*∗*^
Histology	*p*=0.48	*p*=0.87	*p*=0.00001^*∗*^	*p*=0.02^*∗*^	*p*=0.0001^*∗*^	*p*=0.14	*p*=0.07	*p*=0.0005^*∗*^
Grade	*p*=0.23	*p*=0.13	*p*=0.009^*∗*^	*p*=0.05^*∗*^	*p*=0.0001^*∗*^	*p*=0.75	*p*=0.87	*p*=0.0001^*∗*^
Depth of tumor invasion	*p*=0.08	*p*=0.55	*p*=0.0001^*∗*^	*p*=0.00002^*∗*^	*p*=0.0001^*∗*^	*p*=0.55	*p*=0.06	*p*=0.0004^*∗*^
Fragmentation phenomenon	*p*=0.11	*p*=0.44	*p*=0.000004^*∗*^	*p*=0.003^*∗*^	*p*=0.01^*∗*^	*p*=0.85	*p*=0.84	*p*=0.0001^*∗*^
Tumor emboli in CD34-positive vessels	*p*=0.11	*p*=0.37	*p*=0.0004^*∗*^	*p*=0.00003^*∗*^	*p*=0.0003^*∗*^	*p*=0.89	*p*=0.94	*p*=0.09
Tumor emboli in podoplanin-positive vessels	*p*=0.25	*p*=0.75	*p*=0.005^*∗*^	*p*=0.004^*∗*^	*p*=0.26	*p*=0.21	*p*=0.17	*p*=0.0001^*∗*^
Recurrence	*p*=0.42	*p*=0.09	*p*=0.0005^*∗*^	*p*=0.0001^*∗*^	*p*=0.007^*∗*^	*p*=0.51	*p*=0.09	*p*=0.28

## Data Availability

The data that support the findings of this study are available from the corresponding author on reasonable request.
